# Surgical Emergencies—The Surgery of the Head

**Published:** 1903-09

**Authors:** 


Surgical Emergencies—The Surgery of the Head.—By Bayard Holmes, B. S., M. D., Professor of Surgery in the University of Illinois, etc., Chicago. Pages, 569. Price, cloth, $2.50. Published by D. Appleton & Company. 1903.

This work is written by good authority. It is well illustrated, having some 90 figures that are well executed and appropriate, and 14 plates that also do credit to the volume. No criticism of more than minor importance can be urged against the work. The arrangement of the subject matter is not marked by subjects. For instance, the anatomical description, history, etiology, pathology, prognosis and treatment are all run along in the same type, so that a glance can not show the hurried reader what he is looking for. Otherwise the volume is up to a standard commensurate with the high authority and the importance of the subject. T. J. B.

